# The Effect of Healthy Lifestyle Strategies on the Management of Insulin Resistance in Children and Adolescents with Obesity: A Narrative Review

**DOI:** 10.3390/nu14214692

**Published:** 2022-11-06

**Authors:** Valeria Calcaterra, Elvira Verduci, Matteo Vandoni, Virginia Rossi, Giulia Fiore, Giulia Massini, Clarissa Berardo, Alessandro Gatti, Paola Baldassarre, Alice Bianchi, Erika Cordaro, Caterina Cavallo, Cristina Cereda, Alessandra Bosetti, Gianvincenzo Zuccotti

**Affiliations:** 1 Pediatric and Adolescent Unit, Department of Internal Medicine, University of Pavia, 27100 Pavia, Italy; 2 Pediatric Department, “Vittore Buzzi” Children’s Hospital, 20154 Milan, Italy; 3 Department of Health Sciences, University of Milano, 20142 Milan, Italy; 4 Laboratory of Adapted Motor Activity (LAMA), Department of Public Health, Experimental Medicine and Forensic Science, University of Pavia, 27100 Pavia, Italy; 5 Department of Biomedical and Clinical Science, University of Milano, 20157 Milan, Italy; 6 LUNEX International University of Health, Exercise and Sports, 50, Avenue du Parc des Sports, 4671 Differdange, Luxembourg; 7 Neonatal Screening and Metabolic Disorders Unit, V. Buzzi Children’s Hospital, 20154 Milan, Italy

**Keywords:** insulin resistance, obesity, children, adolescents, healthy lifestyle, exercise, diet, nutritional

## Abstract

Childhood obesity is characterized by an increased risk of several metabolic derangements including insulin resistance (IR). The strongest recommendations to prevent obesity and related complications are a balanced and adequate diet and practicing physical activity from early childhood. In this review, we propose to present the effects of healthy lifestyle strategies, including physical exercise and dietary approaches, on the management of IR and related metabolic derangements. All types of exercise (aerobic, resistance and combined training) effectively reduce IR in pediatric patients with obesity; it seems that aerobic and combined training stimulate greater improvements in IR compared to resistance training. Balanced normocaloric or hypocaloric dietary approaches are also valid strategies to address IR; it is not possible to assess the long-term impact of varying macronutrients on cardiometabolic risk. The glycemic index/load evaluation is a useful dietary approach to glucose metabolism control. Similarly, they should adopt the principle of the Mediterranean diet. Randomized studies with longer monitoring are needed to define the benefits of nutritional supplementation on IR. Considering that healthy style acquisition could track to later ages, programs of healthy lifestyle starting with children offer a better preventive strategy to preserve metabolic control and children’s health.

## 1. Introduction

Childhood obesity is characterized by an increased risk of several metabolic derangements including insulin resistance (IR), which is a major link between obesity and other metabolic and cardiovascular complications [1,2]. IR is distinguished by insulin’s reduced ability to stimulate glucose utilization by muscle and adipose tissue and to suppress hepatic glucose production and output [2,3]. It also causes a resistance to insulin’s action on protein metabolism and lipid metabolism and vascular endothelial function and gene expression [2,3].

Several factors are implicated in the etiology of IR, including both genetic and environmental origins [3,4]. The genetic component seems to be polygenic and several genes have been suggested as potential candidates [2]. Other factors capable of influencing insulin sensitivity include obesity, ethnicity, sex, perinatal factors, puberty, sedentary lifestyle, and diet [3].

Adipose tissue, which produces numerous metabolites, hormones and adipocytokines capable of influencing insulin action, plays a key role in IR pathogenesis [3,5,6]. Even fat distribution seems to be related to inflammation severity and occurrence of IR. In fact, visceral fat, compared with subcutaneous fat, is significantly more correlated with poor insulin sensitivity and a higher level of inflammation [2,3].

In children with obesity, diet composition could be an additional factor that may promote as well as worsen IR. Several studies have suggested that a high energy intake and a high-fat, high-carbohydrate, low-fiber diet might increase the risk of developing IR [2,3,4].

Strategies to prevent obesity and IR should be implemented in the early stages of life [3,4]. Among the strongest recommendations from international societies are a healthy and balanced diet, adequate caloric intake, and practicing physical activity from early childhood [2,3]. Indeed, a well-balanced diet and increased physical activity are generally the cornerstones of the treatment of obesity and insulin resistance in children and adolescents. The benefits of different nutritional approach and types of physical exercise (PE) has been also described [2,3].

In this review, we propose to present the effects of healthy lifestyle strategies, including exercise and dietary approaches, on the management of IR and related metabolic derangements in children and adolescents with obesity. Preventive programs of healthy lifestyle starting in childhood may be useful to preserve children’s health.

## 2. Methods

We proposed a narrative review [7], presenting a non-systematic revision of available literature on the topic of healthy lifestyle strategies to manage IR in children and adolescents with obesity. To refine the aim of the narrative review, the most relevant original scientific papers, clinical trials, meta-analyses and reviews published on a specific topic up to September 2022 in the English language, were considered. Case reports or series and letters were excluded. The authors assessed the abstracts of the available literature (*n* = 326) and reviewed the full texts of potentially relevant articles (*n* = 212) that were analyzed to provide a critical discussion. The reference list of all articles was also checked to identify relevant studies. The following search keywords, alone and/or combined, were included: obesity, diet, nutritional supplementation, physical activity, exercise, insulin resistance, inflammation, adolescents, children, multidisciplinary approach, lifestyle. PubMed, Scopus, EMBASE and Web of Science were used as electronic databases for research purposes.

The contributions were independently collected by V.R., C.B., G.M., G.F., P.B., A.B., E.C., C.C. (Caterina Cavallo) and critically analyzed by V.C., M.V., E.V., C.C. (Cristina Cereda), A.B. The resulting draft was discussed by V.C., M.V, E.V. and critically revised by V.C., M.V, E.V., G.Z. All authors approved the final version.

## 3. Childhood Obesity, Insulin Resistance and Adipose Tissue

### 3.1. Childhood Obesity and Insulin Resistance

Childhood obesity is currently a major health problem worldwide. According to the World Health Organization (WHO), nearly 41 million children below the age of 5 and over 340 million children and adolescents aged between 5 and 19 years presented overweight or obesity [2].

Pediatric obesity is a multisystem condition that has several complications, including hyperinsulinemia and IR, hypertension, dyslipidemia, endothelial dysfunction, polycystic ovarian syndrome (PCOS), and non-alcoholic fatty liver disease (NAFLD) and other diseases which shorten patients’ lifespans [5].

IR is a major link between obesity and other metabolic and cardiovascular complications [1,2,8,9]. Pediatric patients with overweight or obesity present hyperinsulinemia and have approximately 40% less insulin-stimulated glucose compared with children with normal-weight. The IR is closely related to the the risk of type 2 diabetes (T2DM) development [5,10,11,12]. The evolution of impaired glucose tolerance in patients with obesity is connected to the worsening of IR and is an intermediate stage in the natural progression to T2DM.

IR is commonly diagnosed in childhood but there are no clear criteria for its definition or detection because of the lack of measurement standardized methods. The gold standard test for the IR assessment in children is the hyperinsulinemic–euglycemic clamp. However, this test is complex, invasive and needs expert operators, and thus is not manageable for clinical practice. As alternative methods, many surrogates have been developed; some of these are based on the measurement of fasting blood samples and others are derived from the oral glucose tolerance test (OGTT), or rarely, the intravenous glucose infusion in absence of reference values [13,14]. Therefore, for clinical practice, the homeostatic model assessment for insulin resistance (HOMA-IR), Matsuda index and the quantitative insulin sensitivity check index (QUICKI) are frequently used in population screening. In particular, HOMA-IR is more suitable and utilizes a mathematical homeostatic model that considers fasting serum glucose and insulin concentrations. In adults, the threshold limit of HOMA-IR accepted for insulin resistance is 2.5, in children and adolescents it is necessary to correlate values with gender and pubertal status, considering that in the pubertal period, there is a normal increase in IR, which can then normalize at the end of puberty [15,16]. More recently, the triglyceride and glucose (TyG) index has also been proposed as a simple surrogate of IR with high sensitivity in recognizing insulin resistance compared with the HOMA-IR [17].

It is officially recognized that adipose tissue, in a condition of positive energy balance, is the site of modifications and mechanisms that lead to a state of low-grade chronic inflammation and, consequently, to an alteration of the normal metabolic profile. Several immune cells are, in fact, recalled and contribute to a massive release of pro-inflammatory cytokines, already locally secreted by the adipose tissue itself, favoring the onset of various complications [18,19]. Among these, IR is commonly associated with obesity [20,21] and it has been repeatedly shown that chronic inflammation is an important cause of obesity-induced IR [22,23,24,25], although it is important to underline that it is only one of the many mechanisms proposed as causative.

### 3.2. Adipose Tissue and Insulin Resistance

As reported by the American Diabetes Association (ADA), IR can be defined as a condition in which cells’ responses to insulin are reduced with respect to carbohydrates, lipids, and proteins, resulting in elevated blood glucose levels [26]. It occurs in various tissues, such as liver, muscle, and adipose tissue (AT), where there is an impairment of the insulin signaling pathway in insulin-reactive cells (hepatocytes, myocytes, adipocytes and β cells) [20,21].

Under physiological conditions, insulin, an anabolic hormone secreted by β cells located in the pancreatic islets, binds to its receptor, causing the latter to autophosphorylation in its Tyr residues and the consequent activation of the tyrosine kinase activity [27,28]. Subsequently, insulin receptor substrates (IRS) are recruited and phosphorylated. This allows for the formation of binding sites for intracellular molecules containing SH_2_ domains. The resulting signaling cascade then leads to the activation of Akt which, in turn, induces the translocation of GLUT-4 and the synthesis of glycogen [20,21]. Inflammation inhibits IRS-1 and the insulin receptor itself [29,30], altering the physiological process.

However, it seems that it also induces other mechanisms that favor the onset of obesity-induced IR, such as: inhibition of the activity of PPARγ, the nuclear receptor responsible for the synthesis of lipids and their storage in cells [30,31]; the increase in free fatty acids (FFA) by stimulating lipolysis and blocking the synthesis of triglycerides (TG) [32]; activation of the NF-kB pathway, responsible for the synthesis of potential IR mediators; and, finally, activation of the c-Jun NH_2_-terminal kinase (JNK) pathway, which interferes with normal IRS-1 phosphorylation and insulin signal transduction [25,33,34,35,36]. It has been observed through studies on mouse models that the genetic inactivation of these two pathways improves insulin resistance [35,37,38,39], a result also confirmed by pharmacological inhibition of the NF-κB pathway with high doses of salicylates or aspirin [37,40].

It is now recognized that obesity predisposes people to the onset of a high local infiltration of macrophages (ATM). Initially, in fact, there is a release of adipokines such as the monocyte chemotactic protein-1 (MCP-1), tumor necrosis factor α (TNF-α), interleukin 1 β (IL-1β) and interleukin 6 (IL-6).

MCP-1 recruits monocytes [41,42,43], which differentiate into macrophages [44,45]. In obesity, the polarization state of macrophages is oriented towards M1 [46], a phenotype characterized by the secretion of several different cytokines, such as TNF-α, IL-1 β, etc. They can potentially cause IR locally, through paracrine mechanisms in the skeletal muscle and in the liver, through endocrine mechanisms [47,48,49] by decreasing the expression of molecules involved in the insulin signaling pathway [50], or by inhibiting the activation of Akt by biosynthesis of ceramide [51]. It has been shown that the accumulation of lipids in the liver and muscle correlates with a high accumulation of fatty acid intermediates such as ceramide [52] and that the pharmacological inhibition of this molecule seems to protect rodents from obesity-induced insulin resistance [53]. Moreover, several studies conducted on animal and humans have shown that weight loss reduces the number of ATMs in the adipose tissue and that this event induces an improvement in IR [41,54], which supports the idea that ATMs are involved in the onset of obesity-induced IR.

Among the other cell populations that seem to play a role in the onset of IR, the studies revealed that obesity increases the number of B cells in the AT and that treatment with a neutralizing antibody improves IR. However, further studies in Rag1^-/-^ knockout mice fed on a high fat diet (HFD) suggests that they require T lymphocytes to induce the occurrence of this condition [55,56,57]. It is known that RAGs, recombination-activating genes, play a role in the recombination and rearrangement of TCRs in T cells and immunoglobulins in B cells and that the deletion of RAGs involves the loss of both T cells (including NKT cells) and B cells. Therefore, *Rag* knockout mice are used for T and B cell studies.

Two other studies have shown how, in conditions of obesity, the level of neutrophils also increases and how the inhibition of neutrophil elastase (NE), a molecule contained in the granules of neutrophils, through genetic deletion or pharmacological inhibition positively correlates with an improvement in IR [58].

Eosinophils are normally present in low concentrations in the AT (~20,000/g of fat), but the number tends to further decrease in conditions of obesity. What has been observed is that an increase due to IL-5 overexpression or helminth infections appears to improve IR [59]. However, it should be noted that this increase occurred in conjunction with changes in body weight and adiposity; therefore, it is not yet clear whether the eosinophil-mediated regulation of insulin resistance is due to direct effects or secondary effects to the changes mentioned above.

Obesity then operates a reduction in circulating Tregs. According to a study that used Foxp3-DTR transgenic mice (toxic receptor for diphtheria), the depletion of Tregs led to a spontaneous development of IR and to an alteration of insulin signaling in vivo in sensitive tissues, including AT. Conversely, their expansion using the IL-2/anti-IL-2 antibody complex in HFD-fed mice improved obesity-induced insulin resistance [60]. A study conducted by Cipolletta et al. has also shown that treatment with thiazolidinediones (TZD) can also improve IR by targeting AT Treg [61].

The roles of NKT cells in obesity have also been considered, with contradictory outcomes. In fact, mice showed improved, exacerbated, or equivalent IR phenotypes without a clear motivation [62,63,64,65,66,67].

Among the secreted cytokines, TNF-α has been observed to produce changes in the transcription of different molecules, in particular the insulin receptor, IRS-1 and GLUT-4, altering the normal insulin signaling pathway [50,68], and treatments aimed at blocking TNF-α improve obesity-induced IR [69,70]. Furthermore, treating obese cells or animals with IL-1β has been observed to induce insulin resistance by inhibiting the insulin signaling pathway [71,72]. On the contrary, the infusion of obese animals with a neutralizing antibody against IL-1β seems to improve IR. However, a recent clinical study of Canakinumab, a humanized monoclonal antibody against IL-1β, revealed that blocking IL-1β does not improve glycemic control in T2DM patients, despite leading to a reduction in inflammation [73]. Therefore, it is important to emphasize that further studies are needed to determine the role of this molecule. Obesity also produces a decrease in the expression of anti-inflammatory factors. Adiponectin has a protective action on fat and glucose metabolism in various tissues [74]. It has in fact been reported that the presence of this molecule positively correlates with insulin sensitivity [75] and that a high percentage in plasma protects the subject from the development of IR and T2DM [76]. In conditions of excess weight, this molecule undergoes a reduction in concentration, due to the suppressive activity of inflammatory factors such as TNF-α and IL-6, [77]. In contrast, it has been observed that peroxisome proliferator-activated receptor gamma (PPARγ) agonists induce the expression of adiponectin, thus also playing a role in promoting insulin sensitivity [78]. Similarly, IL-10 also appears to positively correlate with insulin sensitivity. In fact, IL-10 can prevent insulin resistance induced by a high-fat diet (HFD) in mice by inhibiting the action of macrophages and cytokines [79]. Furthermore, in humans, a reduced production of this cytokine is associated with metabolic syndrome and T2DM [80].

## 4. Exercise Benefits on Insulin Sensitivity

### 4.1. Benefits of Exercise on Insulin Resistance

Exercise is defined as planned, structured, and repetitive physical activity which has as a final or an intermediate objective the improvement or maintenance of PE [31].

According to the Centers for Disease Control and prevention (CDC), the need for pediatric PE depends on a child’s age. Children 3–5 years old are recommended to be active throughout the day for proper growth and development, while for children and adolescents from 6 to 17 years old at least 60 min of moderate to vigorous intensity PE are suggested every day [81]. PE for children can include play, games, sports, recreation, and physical education, as well as planned exercise or training sessions [82].

The lifestyle changes (i.e., healthy eating habits and PE) remain the recommended interventions to adopt for treating pediatric obesity [83]. The WHO [84], in its 2020 guidelines, recommended that children and adolescents undertake at least 60 min of daily moderate- to vigorous-intensity activities to achieve health benefits and prevent the risk of developing obesity. Besides amelioration of general fitness and supporting weight loss, exercise has been associated with multiple positive effects on the health of children and adolescents with overweight or obesity, such as improved insulin sensitivity, cardiovascular and endothelial function, lower low-density lipoprotein levels and higher high-density lipoprotein levels, and reduced risk of morbidity and mortality [11,85,86].

At least two types of PE have to be distinguished: aerobic and resistance training. Aerobic exercise is a moderate-intensity physical exercise, for approximately 60 min, with the purpose of reducing body fat and improving the body’s ability to transport and utilize oxygen in the skeletal muscle and in the heart [87,88]. Running, cycling, or jump rope are considered aerobic exercises. Resistance training, instead, is a short-term strength exercise, in which repetitions of weight or free weight loads are performed to increase lean body mass [87,88]. Sometimes the effects of both types of exercise can enhance the health status of young people with obesity. In fact, it has been demonstrated that an exercise training program utilizing rope jumping was also effective to increase lean body mass, while decreasing abdominal adiposity in adolescent girls with prehypertension [89]. In line with this, in another study on adolescent females with obesity, plasmatic glucose, insulin, and HOMA-IR significantly improved after a 12-week jump rope exercise program [90].

Several studies compared high-intensity interval training (HIIT) with moderate-intensity continuous training (MICT). Twelve-week HIIT was reported to be more effective than MICT for increasing cardiorespiratory fitness (measured as VO_2peak_) in children with obesity, but neither HIIT nor MICT were effective for adiposity, assessed by magnetic resonance imagining [91]. Moreover, no changes were observed concerning plasmatic levels of glucose, insulin, HOMA-IR, triglycerides, and ferritin [91]. In contrast, Meng and collaborators reported beneficial effects of 12-week HIIT or MICT in adolescent boys with obesity. In fact, visceral adipose tissue significantly decreased in the HIIT group, while a significant decrease in body fat percentage was found in the MICT group. However, HOMA-IR significantly decreased in both groups when compared to inactive boys with obesity [92]. A meta-analysis on the effect of HIIT in pediatric obesity was performed by Zhu and colleagues, which concluded that HIIT had positive effects on cardiometabolic risk factors, such as fasting glucose, HOMA-IR, and triglycerides [93]. In another study, children with obesity, both males and females, were subjected to 14-week PE training in combination with nutritional counselling, aimed at increasing the intake of fresh fruits and vegetables, while decreasing soft drinks and fast food [94]. The main finding of this study was the improvement of body BMI and biochemical markers (transaminases and HOMA-IR).

Due to the relevant influence of exercise on IR in children with obesity, several researchers started to analyze and compare the benefits of different types of exercise on these parameters.

Van der Heijden et al. showed that 12-week aerobic training reduced hepatic and visceral fat accumulation, and decreased IR in youth with obesity. Aerobic exercise leads to higher glucose uptake by muscle cells, hence promoting glucose oxidation [33]. Furthermore, this process regulates glucose and insulin release [24], and a reduction in insulin level results in reduced IR [23]. Additionally, resistance training was proven to be effective in improving insulin sensitivity in children; Chang et al. [12] and Shaibi et al. [95] demonstrated that 16 weeks of resistance training, respectively, decreased fasting insulin levels and improved insulin sensitivity in children and adolescents with obesity. The improvement caused by resistance training on insulin sensitivity is induced by the increased muscle contractions, which temporarily increase glucose uptake, and the increased skeletal muscle mass also provides an increased metabolic “reserve” for glucose disposal.

Several studies [96,97,98] found a positive effect of combined training (aerobic + resistance) by decreasing fasting insulin levels and increasing IR and insulin-stimulated glucose disposal. In fact, Bell et al. [99] reported that an 8-week period of combined training was correlated with an improvement in insulin sensitivity (22.2%) in youth with obesity.

A systematic review by Marson et al. [11] suggested aerobic training resulted in superior beneficial effects on insulin sensitivity when compared to resistance or combined (aerobic + resistance) training modalities. However, the number of studies included in the systematic review that were focusing on resistance or combined training interventions was comparatively lower than that on aerobic training. While resistance training still remains controversial, aerobic and combined training interventions have more recently shown comparable results [97].

Although exercise provides multiple health benefits for children and adolescents with obesity, the main problem in this population is the promotion of an active lifestyle and adherence to exercise programs. In fact, children with obesity tend to have a lower perception of their ability to perform exercise [100,101] and this decreased self-confidence leads children and adolescents to avoid highly competitive activities or settings. For this reason, several studies have investigated new modalities to increase adherence to exercise, and some studies highlighted the efficacy of exergames and online supervised training in lowering the dropout rate and promoting a healthy lifestyle compared to regular training modalities in children with obesity [102,103]. These last considerations may represent a starting point to encourage further research in innovative training interventions to promote exercise in children and adolescents with obesity. Additionally, due to ambiguity about what type (aerobic, resistance, or combined) and modalities (regular, exergames, or online) of exercise provide the best results in insulin sensitivity, new research should focus on establishing and clarifying the most effective exercise program for improving insulin sensitivity in youth with obesity.

### 4.2. Molecular Effects of the Exercise on Insulin Resistance

From the molecular point of view, the mechanisms by which insulin sensitivity is ameliorated by PE are several. For example, glucose transporter-4 (GLUT-4), needed for glucose uptake in skeletal muscle and stimulated by insulin secretion, is induced by muscle contraction during PE in an insulin-independent manner. Animal studies revealed that rats subjected to 8-week exercise not only increases GLUT-4 translocation but also the activity of this receptor is increased up to 100-fold [104]. Furthermore, human muscle biopsy samples collected following PE revealed GLUT-4 translocation to plasma membrane [105]. Several studies reported that PE regulates the transcription factor peroxisome proliferator-activated receptor (PPAR) γ coactivator 1a (PGC-1α) and its downstream genes [106]. Activating PPARγ, PGC-1α is able to reduce the lipid entry into skeletal muscle, while also interacting with PPARα, PGC-1α increased fatty acid oxidation genes. Among the several functions mediated by PGC-1α, mitochondrial biogenesis also has to be accounted for. In fact, while mitochondrial dysfunction is linked to IR, PE has been demonstrated to enhance mitochondrial biogenesis, size, number, and oxidative capacity have been observed to be higher following PE [107]. Besides recovering oxidative stress by recovering mitochondrial function, PE is able to decrease inflammation by reducing inflammatory markers and modulating the adipokine profile. In more detail, TNF-α and IL-6 have been implicated in Ser/Thr phosphorylation kinase activation, IRS-1, GLUT-4 and PGC-1α decrease, aggravating IR [108]. On the contrary, plasmatic TNF-α and IL-6 levels decreased after aerobic exercise in children with obesity and overweight [97,109,110]. The adipokine trend is similar to that observed for inflammatory cytokines. Since the increase in leptin and the decrease in adiponectin, the leptin/adiponectin ratio has recently been proposed to be a better diagnostic marker than leptin and adiponectin alone in adolescents [111]. In a group of adolescent females with obesity, the leptin/adiponectin ratio significantly decreased after 12 weeks of high-intensity training, and the decrease was even greater in high-intensity training combined with plyometric exercises [112]. Another important factor to consider is epigenetics. It is also well established that several genes involved in metabolism (PGC-1α and PPARδ) are subjected to DNA or histone modifications (i.e., methylation, demethylation, acetylation, acetylation) and that these modifications could be reversed following PE [113]. In addition to these more frequently studied epigenetic changes, other molecules are under investigation, such as long non-coding RNAs (lncRNAs) and micro-RNAs (miRNAs). Comparing public RNA-Seq data of skeletal muscle biopsies collected from young subjects undergoing different training programs, Bonilauri and Dallagiovanna identified several differentially expressed lncRNAs involved mainly in collagen fibril fusion, skeletal muscle contraction and angiogenesis. Since there were few common deregulated lncRNAs among the exercise type, they concluded that the lncRNA expression pattern was distinct for the different training programs [114]. Interestingly, the miRNA profile is dynamic and its alteration can change significantly during the acute response and the chronic adaptation to exercise [115].

**Figure 1:**
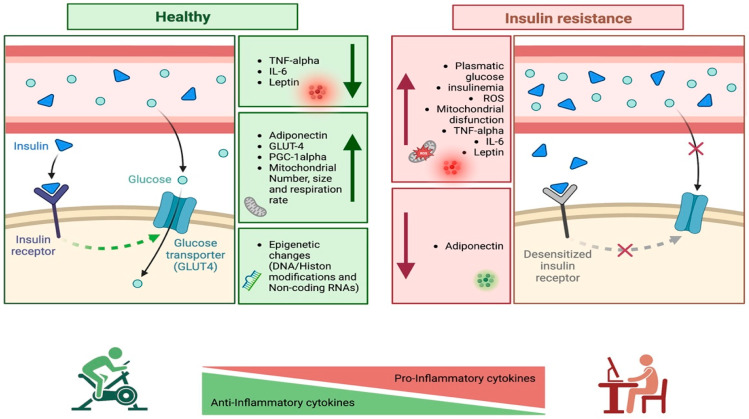
Inflammation, exercise and insulin resistance (created using BioRender).

The relationship between inflammation, exercise and IR is schematized in Figure 1.

## 5. Nutritional Strategies for Modulation of Insulin Resistance

### 5.1. Dietary Approach

Lifestyle interventions, including dietary modification strategies, have been considered the cornerstone of weight management in children and adolescents [116,117]. At the same time, they are attractive as they produce notable health benefits in cardiometabolic outcome among the pediatric age group [118,119].

Among dietary patterns, over the last decades great attention has been paid to the Mediterranean diet (MedDiet), as a balanced nutritional pattern associated with increased metabolic health status in children and adolescents [120]. MedDiet is characterized by the high intake of vegetables, fruits, legumes, and cereals (mainly in unprocessed forms); the low intake of meat and meat products and low to moderate intake of dairy products; the moderate to high intake of fish; and the high intake of unsaturated added lipids, particularly in the form of olive oil [121].

MedDiet has been evaluated as a potential intervention strategy to reduce IR in children. Gallardo-Escribano and colleagues investigated the role of a lifestyle program, which also included a MedDiet intervention, in a group of metabolically healthy prepubertal children with obesity. After 12 months, the population showed statistically significant improvements in the glycemic profile, since reductions in insulin levels and HOMA-IR were observed compared to baseline [122]. Blancas-Sánchez analyzed the efficacy of a nutritional intervention based on the MedDiet in comparison with a healthy standardized diet in school-aged children with obesity or overweight presenting altered HbA1c levels. After 20 weeks, insulin showed a significant change in the intervention group compared to baseline, while no changes were observed within the control group for the same outcome. The only parameter that differed between the groups was insulin levels, which were related to IR [123].

Interestingly, recent studies have pointed out the insulin-sensitizing role of several nutritional components derived from MedDiet foods, namely polyunsaturated fatty acids, anthocyanins, and polyphenols [124]. These constituents provoke modifications in metabolic outcomes of children or adults, by reducing, for example, pro-inflammatory adipocytokines and IR. Moreover, according to nutrigenomic studies, some of these compounds positively affect gene transcription patterns associated with glucose and lipid metabolism [124,125], and this paralleled the beneficial effects on glucose and HOMA-IR in healthy adult subjects [125]. Adherence to a Mediterranean-style diet has also been shown to help to maintain a healthy gut microbiome as well as a better intestinal barrier integrity [126,127,128], which might be altered among children with obesity [129]. These results imply that the MedDiet is a complex and intriguing dietary pattern, with a potential role against IR.

Different types of diet can be adopted to address a specific cardiometabolic risk in pediatric age group. First-line treatment in the presence of obesity and related comorbidities is an intervention based on behavioral counselling and nutritional recommendations to establish balanced daily eating habits [130,131,132,133]. However, at a second stage it may be necessary to implement intervention with an individualized balanced dietary plan with energy restriction for school-aged children [131,132,133]. As reported in the systematic review and meta-analysis of Ho et al., the standard dietary intervention in the presence of obesity and related cardio-metabolic alterations is a hypocaloric balanced diet. This dietary approach implies different ranges of energy restriction, depending on age target, and a macronutrient distribution approximately equals to 50–60%En from CHO, 15–20%En from proteins and <30%En from fats [130]. Several clinical trials have been reporting the positive effects of this approach on several indicators of glucose metabolism, including HOMA-IR, insulin levels, adiponectin, and resistin, in children and adolescents with obesity [134,135,136].

The use of intensive dietary interventions is an emerging area of research and practice, particularly in post-pubertal adolescents with related comorbidities. A recent metanalysis evaluated the use of very low energy diets (VLED), which consists of an energy prescription of approximately 800 kcal/day or less than 50% of the estimated energy requirements, for improving insulin sensitivity and treating T2DM in children and adolescents. Authors reported a significant reduction in fasting insulin concentration post VLED programs [137]. They have shown early short-term success and the possibility of reducing the requirement for medication, however, data are limited to a small pilot study and larger trials are needed [117].

The quality of dietary CHO, particularly glycemic index (GI) and glycemic load (GL) have been evaluated in pediatric obesity and associated risk factors [138,139,140,141,142]. The term GI was introduced in 1981 referring to the area under the blood glucose curve measured two hours after consuming 50 g of test carbohydrates in relation to the results obtained by 50 g of glucose or white bread [143]. Subsequently, the term GL was introduced to quantify the overall glycemic effect of food with respect to its specific carbohydrate content in typically consumed portions [144]. The rationale behind the use of low-GI or low-GL diets is to slow the blood glucose and insulin response following food consumption, as well as increasing satiety and reducing voluntary energy intake [145]. One systematic review compared diets providing a high GI/GL versus low GI/GL in children and adolescents with overweight and/or obesity. Low GI/GL protocols resulted in significantly more pronounced decreases in HOMA-IR [mean difference −0.70, 95%CI −1.37, −0.04] compared to a high GI/GL dietary approach [146].

Recent studies have been evaluating the role of carbohydrate (CHO) content on metabolic outcomes in children with obesity. Kirk et al. randomly assigned a 3-month intervention with low carbohydrate (LC), reduced glycemic load (RGL), or standard portion-controlled (PC) diet to a group of children with obesity aged 7–12 years [147]. Children were followed up until 12 months after intervention. The PC group was instructed to consume age-appropriate portions by means of a calorie-defined meal plan (55–60%En CHO, 10–15%En protein and 30%En fat) with a 500-calorie deficit relative to each subject’s expected energy requirement. Instead, the RGL group was instructed to limit intake of high glycemic GI foods (e.g., white bread, concentrated sugars) by means of a “stoplight approach”. At 12 months, the PC and RGL groups experienced a significant improvement in fasting insulin, while the former also showed an improvement in fasting glucose. On the contrary, the LC group underwent a ketogenic diet with ≤20 g of CHO per day for 2 weeks and, after that, an increase in CHO by 5 to 10 g/week, up to a maximum of 60 g/day, with no limit on energy intake or high protein foods. This group experienced a rapid significant decrease in fasting insulin at 3 months, while at 12 months no significant difference was found compared to baseline. Overall, PC and RGL diets seemed to have a relatively greater effect on lowering fasting insulin and glucose [147]. However, authors concluded that is not clear the degree to which the diet’s macronutrient composition contributed to these variable outcomes because they are undoubtedly also affected by other factors.

The conventional therapeutic approach focuses on a balanced dietary scheme with limited intakes of fat and higher intakes of carbohydrates, and this may not be the preferred option to treat adolescents with obesity and IR [145,148]. A metanalysis compared the efficacy of low-carbohydrate diet compared to conventional balanced diet in pediatric age groups. The dietary intervention with low CHO diet provided 10–20%En from carbohydrates (approximately 20–60 g/day) depending on energy restriction diet, while standard hypocaloric diet provided 50–60%En from CHO and <33%En from fats. Of the five studies that measured insulin levels and/or index of IR, three of them reported improvements following the active intervention regardless the carbohydrate content while two addressed the potential role of a low-carbohydrate diet on insulin levels. Both treatments resulted in an improvement in weight status, thus the author concluded that the primary objective of dietary interventions should be to reduce total energy intake. This means that a low-carbohydrate diet may assist in facilitating improved insulin levels; however, results were reported inconsistently in the evaluated studies and no study included in this review reported follow-up beyond 2 years [149].

Although carbohydrates are the major stimulus for insulin secretion, recent studies have pointed out that they are not the only one [145,150]. In fact, dietary proteins and fats also elicit a significant insulin response [145]. Since GI is only associated with the effect of carbohydrate-containing foods, a new concept of the food insulin index (II) has been proposed [151]. II directly quantifies the postprandial insulin response to any food, not just carbohydrates, with respect to a food reference (glucose). This index has been suggested to be more suitable in evaluating conditions related to insulin exposure, such as obesity [150,151]. Caferoglu et al. were the first to evaluate the postprandial metabolic response and appetite control following the consumption of a meal with the same GI but different II in a group of adolescents with obesity and IR. They showed a significantly lower insulin level at 30 and 120 min after a low-GI/low-II melas compared to low-GI/high-II. Even if meals were matched for GI and macronutrient distribution, they resulted in a less favorable postprandial response when presenting food with a high insulin index. The authors speculate that a low-II diet may reduce nutrient-induced hyperinsulinemia and lead to a greater improvement in IR and insulin sensitivity in adolescents with obesity [150]. These potential implications are of great interest for future research.

Regarding the role of protein content, one RCT investigated the effects of a high protein/low GI (25/45/30En% protein/carbohydrate/fat, GI < 50) compared to a medium protein/medium GI (15/55/30En% protein/carbohydrate/fat, GI ≥ 56) diet in adolescents with overweight/obesity and IR. After 2 years, no significant differences were observed between the two intervention groups regarding IR or parameters of glucose metabolism [152]. Due to poor retention rates and lack of dietary compliance, studies evaluating the effect of protein content on IR are still inconclusive [149,152,153,154].

**Figure 2:**
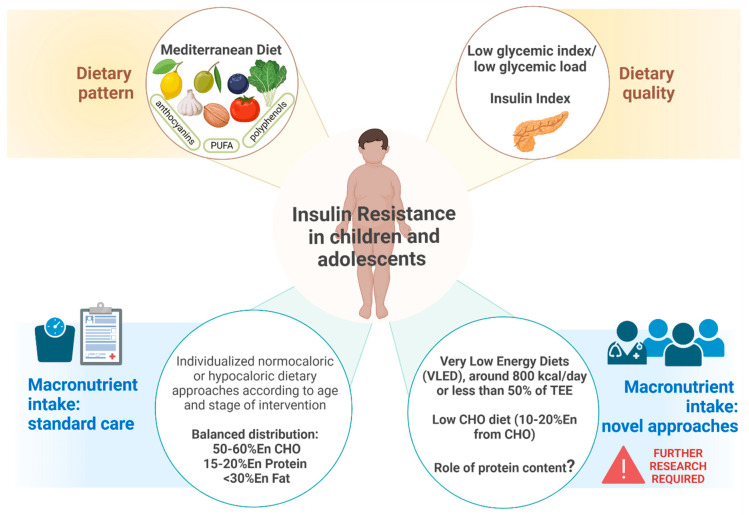
Key impacts of the dietary approach on insulin resistance (created using BioRender).

The key impacts of the dietary approach on IR are schematized in Figure 2.

### 5.2. Nutritional Supplementation

#### 5.2.1. Probiotics

Gut microbiota can modulate the host metabolism directly or through interactions with dietary components; microbial-derived proinflammatory molecules may have direct effects on insulin sensitivity. Gut microbial dysbiosis is a newly emerging environmental risk factor that can modulate insulin sensitivity and risk for T2DM. Microbial dysbiosis in obesity and metabolic derangement is characterized by decreased microbial diversity and richness, increased *Firmicutes* and reduced *Bacteroidetes* in adults and children [155,156].

Dysbiosis leads to an increase in intestinal permeability and triggers the systemic low-grade inflammation in obesity and related disorders. Therefore, modulation of the gut microbiota may have great potential to treat obesity, IR and prevent progression to T2DM. Early interventions aimed at the microbiome, as well as attempts to modify the microbiome may become new opportunities in the prevention and treatment of obesity and carbohydrate metabolism disorders [157,158,159].

Microbiota studies in youth with obesity are limited. A randomized, double-blind, placebo-controlled pilot study with 12 weeks of intervention was conducted at Seattle Children’s Hospital on 8 adolescents with severe obesity (Randomized = 15; Drop out = 7; Placebo = 4; Probiotic = 4), from June 2018 to July 2019. The aim of the study was to determine if an oral multi-strain probiotic (*Lactobacillus casei* + *Lactobacillus rhamnosus* + *Bifidobacteria*) would change the gut microbiota, IR and inflammation in adolescents with severe obesity, by using metagenomic shotgun sequencing. Anthropometry, fasting glucose, insulin, C Reactive Protein and stool for microbiome and calprotectin were collected at baseline (week 0) and 12 weeks after intervention. The study concluded that mean change in fasting glucose was significantly lower in the probiotic group as compared to the placebo group. The gut microbial Firmicutes-to-Bacteroidetes ratio had a greater decline from week 0 to week 12 in the probiotic group but was not statistically significant as compared to the placebo group. Weight and BMI trended to remain stable in the treatment group as compared to the placebo group but were not significant. No significant change in the fasting insulin, HOMA-IR, or serum and stool inflammatory markers were noted between the two groups. One participant in the treatment arm reported adverse effects of gastrointestinal intolerance [160].

*Bifidobacterium breve* has been largely investigated for its beneficial effects, in particular, its ability to reduce chronic inflammation and modulate gut microbiota composition and SCFA production. Solito et al. [161] evaluated the efficacy of two B. breve strains (*B. breve BR03* and *B. breve B632*) to improve glucose and insulin homeostasis and to influence inflammatory status and gut microbiota profile. It was a cross-over, double-blind, randomized control trial conducted on 101 children (6–18 years; M/F 54/47), on dietary training, affected by obesity and IR. The study lasted 8 weeks with 4 weeks washout period. During the analysis, it emerged that all subjects improved in metabolic parameters (BMI, waist circumference, systolic and diastolic blood pressure, HOMA-IR, lipid profile) and decreased weight and Escherichia coli counts. Moreover, the assumption of probiotics improved insulin sensitivity at fasting and during OGTT. Cytokines and GLP1 did not vary. No adverse events were reported [161].

The relationship between gut microbiota and obesity was also investigate by *Bifidobacterium pseudocatenulatum* CECT 7765 administration. The study, published in the *European Journal of Nutrition* in 2019, evaluated the effects of this probiotics strains on cardiometabolic risk factors, inflammatory cytokines and gut microbiota composition in children with obesity and IR, in particular lipid profile, high-sensitive C-reactive protein (hs-CRP), monocyte chemoattractant protein-1 (MCP1—chemokine which trigger the early stage of atherosclerotic plaque formation) and omentin-1 (anti-inflammatory adipokine, produced in response to inflammatory stimuli and implicated in the regulation of insulin sensitivity and endothelial function) [162].

The study included 48 children with obesity and IR (10–15 years old), all patients received dietary advice to ameliorate lifestyle attitude. The probiotic strain was provided in single-dose capsules containing between 1 × 10^9^ and 1 × 10^10^ colony-forming units (CFU) per day. The control group received capsules containing the same ingredients without the bacteria. The study highlighted a significant improvement in BMI and percentage of fat mass in all children after the intervention, suggesting that weight changes are related to the dietary advice. This intervention also demonstrated that the intake of *B. pseudocatenulatum* led to a decrease in circulating hs-CRP and MCP-1 and an increase in HDL-C and omentin-1 in children with obesity and IR compared to the control group, i.e., an improvement in inflammatory status. Finally, probiotic administration significantly increased the proportion of the *Rikenellaceae* family members, particularly of the *Alistipes* genus, associated with lean phenotype [162]. A. Benítez-Páez et al. identified, through a cross sectional study, previously unknown species whose depletion (*Blautia luti* and *Blautia wexlerae*) is associated with IR in individuals with obesity. They analyzed the gut microbiota structure and the cytokine profile in children with obesity with and without IR and the immune regulatory properties of bacterial species linked to metabolically healthy phenotypes. The study included 51 children between the ages of 5 and 17 years and of Caucasian race, *n* = 35 subjects presented obesity, while 16 were children with normal nutritional status. Anthropometry measurements (weight and height), biochemical parameter (glucose, insulin, lipid profile, HOMA-IR), inflammatory marker (IL-6, IFN-γ, TNF-α, MCP-1) and microbiota analysis were evaluated. Results indicated that *B. luti* and *B. wexlerae* species could contribute to the maintenance of intestinal immune homeostasis in metabolically healthy subjects and that their depletion is associated not only with obesity but also with metabolic complications such as IR and related inflammatory markers [163]. The gut microbiome has been shown to be an important part of the metabolic processes. The findings support the notion that microbiome-based interventions in childhood could improve the management of co-morbidities associated with obesity, taking advantage of this window of opportunity for metabolic disease prevention.

#### 5.2.2. Fiber

Fiber can exist as dietary fiber (naturally occurring in food), or functional fiber (added during the processing or preparation of food or consumed separately as a supplement). Fiber can be insoluble or soluble in water. Insoluble fibers include cellulose, hemicellulose, and lignin, whereas soluble fibers include various gum, pectin, β-glucan, oligosaccharide, resistant dextran, and resistant starch. Chitin and chitosan are indigestible amino-polysaccharides that are found in or are derived from the exoskeletons of arthropods [164,165].

Modulation of gastrointestinal transit may be one of the mechanisms underlying the beneficial health effects of dietary fibers. These effects include improved glucose homeostasis and a reduced risk of developing metabolic diseases such as obesity and T2DM [166,167]. Indeed, fiber ingestion contributes to reduced appetite, delaying the colonic transit time, stimulating the satiety hormone and endogenous glucagon-like peptide-1 (GLP-1) secretion [168]. Furthermore, colonic transit has a major impact on the gut microbiota, which may be involved in many physiological functions in energy and substrate metabolism, metabolic cross-organ signaling and insulin sensitivity [169].

In 2003, a review on psyllium fiber supplementation in children with obesity was conducted with the aim to assess the management of abnormalities of carbohydrate and lipid metabolism. After psyllium supplementation, data recruited showed a change in postprandial glucose in T2DM patients, ranged from −12.2 to −20.2%. The reviewed evidence seems to show that psyllium improves glucose homeostasis and the lipid and lipoprotein profile [170].

In 2020, Maffeis et al. aimed to verify whether the intake of Policaptil Gel Retard (PGR) 20 min before meal ingestion, in comparison with placebo, was able to reduce the postprandial lipid and glucose profile as well as ghrelin, insulin and appetite in a group of children with obesity. PGR is a medical device in tablets, composed of polysaccharidic macromolecules (cellulose, hemicellulose, pectin, mucilage) and derived from raw materials (glucomannan, cellulose, Opuntia pulp stem, chicory root and freeze-dried mallow root, flaxseed and linden flower mucilage) rich in fibers. The study (a double-blind randomized clinical trial) included 46 children with obesity (8–12 years), none followed nutritional advice. Two PGR tablets or placebos were given in fasting conditions, before the ingestion of a mixed meal (15 kcal/kg lean body mass). Blood samples were taken at baseline and for 4 h. Blood lipids, glucose, insulin, ghrelin, and glucagon-like peptide-1 (GLP-1) were evaluated. The study revealed that single intake of two tablets of PGR was associated with a significant reduction in appetite, ghrelin, and triglycerides in the postprandial period while blood glucose, insulin, non-esterified fatty acids (NEFA) and GLP-1 profiles were not significantly different between the two groups. The conclusion is that further evaluation of chronic PGR assumptions are necessary to assess the impact on weight and glucose metabolism [171].

In a recent study, a randomized double-blind placebo-controlled trial was conducted on *n* = 155 Thai children with obesity (ages 7–15 years) to determine the effects of inulin supplementation on inflammation, and to assess the relationships of inflammatory cytokines with adiposity and IR. Patients were randomly assigned to inulin (intervention), maltodextrin (placebo), and dietary fiber advice groups. All participants received monthly follow-up and identical advice on lifestyle modification for six visits. In all groups BMI z-score and body fat percentage significantly decreased without significant differences between the three clusters. The authors demonstrated that IL-1β and TNF-α were significantly decreased in all groups, while IL-6 displayed an increase. The study carried out showed the change in both systemic and local inflammations after inulin supplementation and a significant relationship of IL-6 with fat mass index and IR at baseline. In the light of recorded performances, the authors suggest IL-6 as a surrogate marker of inflammation in children with obesity who are at risk for IR and metabolic syndrome. Further studies are needed to confirm these data [172].

Among the types of fiber, chitosan is one of that has received great attention for improving obesity-related markers. A randomized clinical trial was conducted in order to analyze the effects of chitosan supplementation on appetite-related hormones, anthropometric indicators of obesity, and lipid and glycemic profiles in adolescents with overweight or obesity. The study included 61 adolescents with overweight and obesity, who were randomly allocated to receive chitosan supplementation (*n* = 31) or a placebo as control (*n* = 30) for 12 weeks. The supplementation demonstrated an improvement in obesity indicators (weight, body mass index, waist circumference), lipid profile, glycemic markers (insulin, fasting blood glucose HOMA-IR), leptin, adiponectin and neuropeptide Y. The improvement in these parameters in the chitosan supplementation group has been confirmed in the comparison with the placebo cluster [173].

An interesting study was conducted in 2018, in Quebec, Canada, on the assessment of dietary intakes of macronutrients and food groups in the association with insulin sensitivity and insulin secretion over a 2-year period in children with a family history of obesity. The authors focused their attention on dietary fiber and saturated fats, dietary habits were assessed at baseline using three 24 h dietary recalls. Insulin sensitivity was assessed by the Matsuda insulin sensitivity index (ISI) and the homeostatic model assessment of IR. Insulin secretion was assessed at 30 min and at 120 min during an oral-glucose-tolerance test. Results highlight that every additional serving of vegetables and fruit increased the Matsuda ISI and decreased HOMA-IR by ∼2% after 2 years. Strategies aimed at increasing access to an intake of vegetables and fruits may lead to improved insulin sensitivity over time and contribute to the prevention of T2DM [174].

Considering the data published, fiber supplementation may significantly augment high-fiber eating strategies by further promoting satiety and reducing cardiometabolic risk factors. However, evidence linking the intake of dietary fiber and high-fiber foods to the health outcomes in IR are not consistent in children and adolescent with obesity, and the reason may depend on large differences between studies with regard to age, type and amount of dietary fibers, diet composition, metabolic phenotype of the individual and duration of follow-up. Long-term clinical trials looking at the optimal form, quantity, and frequency of dietary fiber supplements are greatly needed to clarify their potential in the long-term management of obesity.

#### 5.2.3. Long Chain Polyunsaturated Fatty Acids

Pediatric obesity and IR represented an inflammatory condition which is potentially reversible. Long-chain polyunsaturated fatty acids omega 3 (LCPUFA-ω3), eicosapentaenoic (EPA), and docosahexaenoic (DHA) exert an anti-inflammatory activity and stimulate the expression of genes involved in the metabolic pathways of insulin action. On the basis of this evidence, PUFA may beneficially influence insulin sensitivity, particularly n-3 PUFA, whereas the n-6 PUFA, arachidonic acid (AA) may have an adverse effect. In particular, a higher dietary n-6:n-3 ratio has been associated with increased IR [175,176].

Burrows T. et al. examined the relationship between the omega 3 index, weight status and IR in a sample of Australian primary-school-age children, classified as without obesity (*n* = 24) or with obesity (*n* = 24) on the basis of body mass index z-score. Fat dietary intake was evaluated using a food frequency questionnaire, erythrocyte composition was determined by gas chromatography, and omega 3 index was investigated by adding eicosatetraenoic and docosahexaenoic acid percentage values. The study demonstrated an association between omega 3 index, obesity and IR in children. A total of 33% of patients with obesity had an omega 3 index defined “high risk” (<4) and in the group without obesity only 17% showed this score. The results indicated that children with obesity had altered erythrocyte fatty acid composition unrelated to reported dietary intake. A moderate, significant correlation was found between omega 3 index and fasting glucose level and HOMA-IR [177].

A similar study was conducted on 56 randomly school-age children with obesity. The aim was to compare the levels of EPA in schoolchildren with obesity and with or without IR. IR was determined by HOMA index and the serum levels of eicosatetraenoic acid were determined by gas chromatography. Results showed that primary-school-aged children with obesity and with IR had lower plasma levels of eicosatetraenoic acid than the non- IR group [178].

A longitudinal study, conducted by Sarah Marth et al. on 705 European children (2–9 years old—subsample of IDEFICS Study), aimed to investigate the association of whole blood n-3 and n-6 polyunsaturated fatty acids (PUFA) with IR. The fatty acid blood profiles were analyzed and included as a weight percentage of all fatty acids detected. IR was determined by HOMA-IR. The association was investigated at baseline and after 2- and 6-year follow-ups using models with basic and additional confounder adjustment as well as beingstratified by sex and weight status. Unfortunately, results do not point to an association between n-3 PUFA or AA and IR [179].

Results from intervention studies are variable, many studies have found small decreases in HOMA-IR or fasting insulin in children with obesity or IR, others reported borderline or no effects.

A double-blind, randomized, placebo-controlled, parallel trial was carried out to evaluate whether supplementation with LCPUFA-ω3 reduces IR and weight in adolescents with obesity. Children were randomly assigned to receive daily doses of 800 mg EPA + 400 mg DHA (*n* = 119) or 1 g sunflower oil as placebo (*n* = 126) for 3 months, together with a hypocaloric diet consisting in 700 kcal restriction from usual daily intake. At baseline, 92% of children presented IR, 66% hypertriglyceridemia, 37% low-grade inflammation, and 32% metabolic syndrome. No effect of LCPUFA-ω3 supplementation on weight, insulin, or HOMA was detected. No effect of supplementation was observed on changes in insulin concentration after considering changes in body weight [180].

In light of evidence suggesting that both metformin and ω-3 can improve the metabolic states of children with obesity, Juarez-Lὁpez et al. focused their work on administering these drugs to IR children with obesity to assess their effects on fasting glucose, insulin, HOMA-IR index, lipid profile, and BMI. They included 201 adolescents with obesity and IR, *n* = 98 received 500 mg of metformin while *n* = 103 obtained 1.8 g of omega 3 PUFA for 12 weeks. No lifestyles intervention was performed. No adverse events were reported. The effects of metformin were observed on weight loss and lipid profile (LDL-c and HDL-c, no effects on triglycerides), the lack of effect on fasting glucose and insulin or HOMA-IR was probably due to the insufficient time and dose used. Children supplemented with omega 3 showed positive weight-reduction effects on fasting glucose and lipid profile (HDL-c and triglycerides). The results of this study suggest that ω-3 may be useful as an adjuvant therapy for children with obesity and IR [181].

#### 5.2.4. Vitamin D

Vitamin D plays a central role in bone metabolism, but it is also involved in glucose homeostasis. Vitamin D promotes the release of insulin, its transcription and its receptor genes and promotes the expression of glucose transporter GLUT4 in muscles and its translocation into adipocytes [182].

A higher incidence of low total vitamin D levels has been reported in children with obesity, compared to the general population, Several mechanisms have been considered to explain the lower concentration of vitamin D in the obesity condition: inadequate diet consumption, decreased sun exposure due to sedentary lifestyle, 25-hydroxy-vitamin D trapping in adipose tissue, and a combination of these factors [183,184].

The hypothesis of a direct relationship between vitamin D and insulin sensitivity (IS) and β-cell function (BCF) in a sample of non-diabetic, children with overweight/obesity and adolescents was tested. It featured in a cross-sectional study carried out at the Childhood Obesity Outpatient Clinic, University Hospital of Verona, in which 122 Caucasian children with overweight and obesity were enrolled. Vitamin D insufficiency (20–29 ng/mL) and deficiency (< 20 ng/mL) were found in 50% and 40.2% of children, respectively. The main finding of this study is the significant association between total vitamin D and IS, IR and insulin secretion indices. The authors found that total vitamin D was a significant predictor of IS, IR and BCF, in terms of both HOMA-β and the 2nd phase of insulin secretion, supporting the role of vitamin D supplementation as a potential intervention strategy for the management of IR in children and adolescents with obesity [185]. These results do not align with data which emerged previously in a 12-week double-blind, randomized trial, conducted on 46 Caucasian adolescents with obesity (BMI > 95th percentile). The objective of the study was to determine the effect of two doses of cholecalciferol (vitamin D3) supplementation on insulin action and BCF function in adolescents with obesity. The subjects were randomly assigned to receive either 400 IU/d (*n* = 23) or 2000 IU/d (*n* = 23) of vitamin D3. The results of the current study suggest that vitamin D supplementation, taken even at a dose of 2000 IU/d by nondiabetic and vitamin D–replete adolescents with obesity for 12 weeks, has no effect on insulin sensitivity and pancreatic b-cell function, or on lipid profile [186].

In 2012, in Iran, a triple-masked controlled trial was conducted to investigate the effects of oral vitamin D supplementation on IR and cardiometabolic risk on children and adolescents with obesity. Participants (10–16 years old) were randomly assigned into two groups: *n* = 21 received oral vitamin D (300.000 UI) and *n* = 22 received a placebo for 12 weeks. In the intervention group, a significantly reduction in serum insulin, triglyceride concentrations and HOMA-IR index was observed, both when compared with the baseline and with the placebo group. No significant variation was observed in lipid profile [187].

Bilici et al. evaluated the effect of 2000 IU/day vitamin D supplementation on IR and cardiovascular risk parameters in vitamin D deficient adolescents with obesity. A total of 96 patients (10–18 years old) were enrolled in the study, 56.2% of cases had vitamin D deficiency while the other 43.8% of cases had a sufficient vitamin D level. The supplementation duration in cases with vitamin D deficiency was 3 months, only 23 cases receiving the treatments regularly and were evaluated at the 3rd month of the supplementation. After intervention, vitamin D levels had reached up to sufficient levels with the treatment in 95.6% of the vitamin D deficient cases. None of the cases had hypercalcemia during the treatment period. At the end of the 3 months, total cholesterol and LDL cholesterol (cardiovascular risk parameters) had decreased and the level of IL-6 had reduced, while insulin, glucose and HOMA-IR had no significant changes [188].

A similar study was conducted in 2014 with the aim of determining the effects of vitamin D3 supplementation on 25-hydroxyvitamin D, lipid profile and markers of IR in adolescents with obesity. It was a double-blind, randomized, placebo-controlled trial where 58 adolescents with obesity received either vitamin D3 (2000 IU/day) or a placebo for 12 weeks. Forty-four children completed the study. Vitamin D3 supplementation resulted in a modest increase in 25(OH)D concentration in adolescents with obesity, but did not affect the lipid profile and markers of IR and inflammation [189].

Belenchia et al. determined the impact of treatment with vitamin D in American children and adolescents with obesity (with 25-hydroxy vitamin D level < 20 ng/mL) on vitamin D levels, insulin secretion and sensitivity. Patients were randomized to receive either 50,000 IU vitamin D_2_/week or a placebo for 12 weeks. Lipid profile, calcium levels, glucose tolerance and insulin levels were evaluated before/after intervention. The supplementation significantly improved serum 25(OH)D levels, however, significant changes in insulin secretion and sensitivity were not observed in either group [190,191].

An interesting work was conducted in Florida at the Nemours Endocrinology and Metabolism Clinic in Jacksonville. A total of 39 prepubertal subjects were enrolled. The aim of the study was to determine the effects of a placebo vs. an encapsulated supplement of fruit and vegetable juice concentrate on serum β-carotene levels, IR, adiposity, and subclinical inflammation in boys. Patients were randomized for the intervention, the duration of which was 6 months. After supplementation, HOMA-IR decreased in the boys with overweight as well as the abdominal fat mass [192].

Data published suggest that a periodic monitoring of vitamin D concentration in children and adolescents with overweight and obesity may be justified as well as the treatment with 25(OH)D in subjects with vitamin D deficiency or insufficiency. Indeed, larger scaled prospective randomized studies with longer follow-up periods are needed to obtain more informed outcomes on IR and cardiometabolic risk.

Most relevant literature on the effects of nutritional supplementation on insulin resistance are summarized in Table 1.

## 6. Conclusions

There are several demonstrations supporting the role that the inflammatory state induced by obesity plays in the onset of IR. Therefore, preventive strategies such as exercise and dietary approach may be useful to mitigate the inflammatory process and to reduce the risk of obesity-related complications.

Exercise is a crucial component of a healthy lifestyle, it helps achieve better health outcomes and reduces IR in children and adolescents with obesity. The current literature review showed that all types of exercise (aerobic, resistance and combined training) effectively reduce IR in children and adolescents with obesity. Nevertheless, it is unclear what type of exercise is the most effective, but it seems that aerobic and combined training stimulates greater improvements in IR compared to resistance training. Further studies should focus on establishing what type of exercise provides greater improvements in insulin sensitivity in children and adolescents with obesity. Overall, the dietary approach is an important strategy to address IR among pediatric-age-children. Children with metabolically unhealthy obesity might benefit more from these interventions, considering the already existing alterations in the indicators of glucose metabolism. Depending on the stage of intervention and the age of the child, balanced normocaloric or hypocaloric dietary approaches are valid strategies. To date, it has not been possible to assess the long-term impact that varying the macronutrient content of the diet has on cardiometabolic risk, including IR. It may be appropriate to evaluate a low-carbohydrate diet for the treatment of IR in children and adolescents; however, further research is required before recommendations can be made. The role of carbohydrates’ quality has increasingly been reported in dietary intervention strategies for children with metabolic alterations. The evaluation of GI and LG is a useful dietary approach and patients should be educated to recognize the different impacts that foods have on indicators of glucose metabolism. Similarly, they should adopt the principle of the Mediterranean diet, recognized as a potential strategy for the treatment of obesity and related comorbidities. Lastly, future studies on insulin index diets may assist in developing new dietary interventions for adolescents with obesity and IR, but this assumption needs to be confirmed by further clinical trials. Prospective randomized studies with longer monitoring periods are needed to define the exact role of nutritional supplementation and microbiome-based interventions on IR.

Considering that healthy style acquisition could track to later ages, programs of healthy lifestyle starting in childhood offer a better preventive strategy to preserve metabolic control and children’s health.

## Figures and Tables

**Table 1:** Most relevant literature on the effects of nutritional supplementation on insulin resistance.

Supplementation	Type of Study	Subjects	Intervention	Intoleranceand/or Side Effect	Observed Effects
Probiotics	DB—placebo—RCT [160]	Randomized = 15 Drop out = 7 Placebo = 4 Probiotic = 4 Age: adolescents	Supplementation: Multi oral multi-strain probiotic: *Lactobacillus casei* + *Lactobacillus rhamnosus* + *Bifidobacteria* No Dietary adviceDuration: 12 weeks	Gastrointestinal intolerance: 1 patients	- Mean change in fasting glucose was significantly lower in the probiotic group as compared to the placebo group - Gut microbial Firmicutes to Bacteroidetes ratio had a greater decline from week 0 to week 12 in the probiotic group but was not statistically significant as compared to in the placebo group - Weight and BMI tended to remain stable in the treatment group as compared to the placebo group but was not significant. - No significant change in the fasting insulin, HOMA-IR, or serum and stool inflammatory markers were noted between the two groups
	CO-DB-RCT [161]	101 children affected by obesity and IR Age: 6–18 years	Dietary advice Supplementation: *B. breve BR03* + *B. breve B632* or placebo Duration: 8 weeks with 4 weeks wash out period	No adverse events	- All subjects improved metabolic parameters (BMI, waist circumference, systolic and diastolic blood pressure, HOMA-IR, lipid profile), decreased weight and Escherichia coli counts - Probiotics assumption improved insulin sensitivity at fasting and during OGTT. - Cytokines and GLP1 did not vary.
	RCT [162]	48 children with obesity and IR Age: 10–15 years	Dietary advice Supplementation: *Bifidobacterium pseudocatenulatum* CECT 7765 strains or placebo Dose: single-dose capsules containing between 1 × 10^9^ and 1 × 10^10^ colony forming units (CFU) per day Duration: 13 weeks	No adverse events	- Improvement in BMI and percentage of fat mass in all children - ↓ hs-CRP and MCP-1 - ↑ HDL-c and omentin-1 - Improvement in inflammatory status - ↑ *Rikenellaceae* family members
	RCT [163]	51 children Age: 5–17 years N° = 35 obesity N° = 16 normal nutritional status	Microbiota analysis Duration: not indicated	No adverse events	- *B. luti* and *B. wexlerae* species could contribute to the maintenance of intestinal immune homeostasis in metabolically healthy subjects - ↓ *B. luti* and *B. wexlerae* species associated not only with obesity but also with metabolic complications: IR and related inflammatory markers
Fiber	DB-placebo-RCT [171]	46 children with obesity Age: 8–12 years	No dietary advice Intervention: Policaptil Gel Retard (PGR) or placebo: 2 PGR tablets or placebo were given in fasting condition, before the ingestion of a mixed meal (15 kcal/kg lean body mass). Blood samples were taken at baseline and after 4 h. Duration: 1 day	No adverse events	- A single intake of two tablets of PGR was associated with a significant reduction in appetite, ghrelin, and triglycerides in the postprandial period - Blood glucose, insulin, non-esterified fatty acids (NEFA) and GLP-1 profiles were not significantly different in the two groups
	DB-placebo-RCT [172]	155 Thai children with obesity Age: 7–15 years	Dietary advice Low-energy and low-fat diet 1° Group: 13 g of isocaloric oligofructose enriched inulin extracted from the Thai Jerusalem artichoke daily at 30 min prior to dinner 2° Group: 11 g of isocaloric maltodextrin 3° Group: dietary fiber advice group Duration: 6 months	No adverse events	- In all groups BMI z-score and body fat percentage significantly decreased without significant differences between 3 clusters - ↓ IL-1β and TNF-α - ↑ IL-6 - Significant relationship between IL-6 with fat mass index and IR at baseline
	DB-RCT [173]	61 adolescents with overweight and obesity Age: 10–19 years	Chitosan supplementation (*n* = 31): 1.5 g (twice a day a totalof 3 g) Placebo (*n* = 30): maltodextrin Timing: daily 30 min to 1 h before lunch and dinner Duration: 12 weeks	No adverse events	- Improvement of weight, BMI, waist circumference, lipid profile, insulin, fasting blood glucose HOMA-IR, leptin, adiponectin and neuropeptide Y
Lcpufa	Observational Study [177]	24 children without 24 children with obesity Age: 5–12 years	No intervention	No adverse events	- 33% of patients with obesity had an omega 3 index defined “high risk” (<4) and in group without obesity only 17% showed this score. - A moderate, significant correlation was found between omega 3 index and fasting glucose level and HOMA-IR
	RCT [178]	56 children with obesity Age: mean age was 109.2 months	No intervention	No adverse events	- Children with insulin-resistance had lower plasma levels of eicosatetraenoic acid than the non- insulin-resistance group
	Longitudinal Study [179]	705 European children Age: 2–9 years old—subsample of IDEFICS Study	No intervention Duration: association was investigated at baseline and after 2- and 6-year follow-ups	No adverse events	- No associations were found
	DB- placebo- parallel RCT [180]	245 children Age: 12–18 years	Dietary advice Hypocaloric diet Supplementation: daily doses of 800 mg EPA + 400 mg DHA Placebo: 1 g sunflower oil Duration: 3 months	No adverse events	At baseline: - IR: 92% - ITG: 66% - Low-grade inflammation: 37% - MS: 32% No effect of LCPUFA-ω3 supplementation on weight, insulin, or HOMA No effect on insulin concentration
	RCT [181]	201 adolescents with obesity and IR	No dietary advice Supplementation: N°98 Metformin—Dose: 500 mg N°103 omega 3 PUFA—Dose 1.8 g Duration: 12 weeks	No adverse events	Metformin effects: - weight loss - improvement in LDL-c and HDL-c - no effect on fasting glucose and insulin or HOMA-IR PUFA effects: - ↓ fasting glucose - Improvement in triglycerides and HDL-c
Vitamin d	Cross sectional study [185]	122 Caucasian children with overweight and obesity Age: 12.8 ± 2.3 years	No intervention	No adverse events	- Significant association between total vitamin D and IS, IR and insulin secretion indices
	DB-RCT [186]	46 Caucasian adolescents with obesity Age: 12–18 years	Supplementation: - 400 IU/d or 2000 IU/d of vitamin D3 Duration: 12 weeks	No adverse events	- No effect on insulin sensitivity and BCF, lipid profile
	Placebo—RCT [187]	43 children Age: 10–16 years	Supplementation: - N = 21 received oral vitamin D (300.000 UI) - N = 22 received placebo Duration: 12 weeks	No adverse events	In the intervention group: - Significant reduction in serum insulin, triglyceride concentrations and HOMA-IR index - No significant variation was observed in lipid profile
	RCT [188]	96 children Age: 10–18 years	Supplementation: - 2000 IU/day vitamin D Only 23 cases receiving the treatments Duration: 3 months	No adverse events	- 56.2% vitamin D deficiency - 43.8% sufficient vitamin D level - After intervention: - no significant changes in insulin, glucose and HOMA-IR - ↓ TC, LDL cholesterol, IL-6
	DB-placebo-RCT [189]	44 adolescents with obesity Age: 12–18 years	Supplementation: - Vitamin D3 (2000 IU/day) or placebo Duration: 12 weeks	No adverse events	- Modest increase in 25(OH)D concentration - No effects on lipid profile and markers of IR and inflammation
	DB-placebo-RCT [190,191]	29 children with obesity Age: 13–17 years	Supplementation: - 50,000 IU vitamin D2/week or a placebo Duration: 12 weeks	No adverse events	- Significantly improved serum 25(OH)D levels - No significant changes in insulin secretion and sensitivity
	RCT [192]	39 prepubertal boys	Supplementation: - encapsulated supplement of fruit and vegetable juice concentrate or placebo Duration: 6 months	No adverse events	- ↓ HOMA-IR and abdominal fat mass in overweight boys

## Data Availability

Not applicable.
